# Rare (uro-)genital pathologies in young girls mimicking sexual abuse

**DOI:** 10.1007/s00414-021-02621-z

**Published:** 2021-05-31

**Authors:** Martine Schaul, Thorsten Schwark

**Affiliations:** Service Medico-Judiciaire, Département Médecine Légale, Laboratoire National de Santé, 1, Rue Louis Rech 3555, Dudelange, Luxembourg

**Keywords:** Vaginal bleeding, Child sexual abuse, Urethral prolapse, Perineal groove, Failed fusion of the perineum, Sexual assault

## Abstract

Examinations of young children for suspicions of sexual abuse are challenging for the involved medical specialists because the consequences of the interpretation of the findings can be severe and dramatic. A broad knowledge of differential diagnoses including rare pathologies like urethral prolapse and failure of the midline fusion of the perineum, known as perineal groove, is essential in order to avoid unnecessary diagnostics and treatment, prejudgment, and to reduce patient family’s anxiety. We report two independent cases of girls aged 7 months and 5 years suffering from these rare pathologies, one presenting with painless lower genital tract bleeding, the other showing a lesion of the perineum as random finding during a neuropediatrician’s consultation. In both cases, the pathologies were initially misdiagnosed as injuries due to sexual assault, and judicial investigation procedures were initiated. In this paper, the characteristic symptoms and morphology of urethral prolapse and perineal groove are presented to enhance the awareness of these pathologies among forensic experts and help to establish the correct diagnosis.

## Introduction

After an assumed sexual abuse, medical findings in pediatric gynecological exams are rare, and the proof is generally hard to bring. Even if the victim is examined immediately after the presumed assault, the prevalence of injuries is known to be low [1]. In contradiction to these data, unexplained anogenital symptoms and disorders in a child are frequently leading to suspicions of sexual assault and are causing anxiety to the parents or other caregivers. The physician carrying out the subsequent gynecological examination is frequently responsible for the decision if a police investigation is initiated. Beside the knowledge of typical injuries caused by penetration, a broad expertise regarding differential diagnoses, such as normal variants, infections, and even rare pathologies, e.g., urethral prolapse and failed fusion of the perineum, is essential to avoid mistakes [2–4]. We present two cases of rare gynecological diseases in children that initially led to a police investigation. The demonstration of the typical findings in the respective cases should increase the awareness of these pathologies and enhance early recognition in order to provide adequate care, and avoid repeated examinations, unjustified suspicion of parents, and needless police operations.

## Case reports

### Case 1: history

**Fig. 1:**
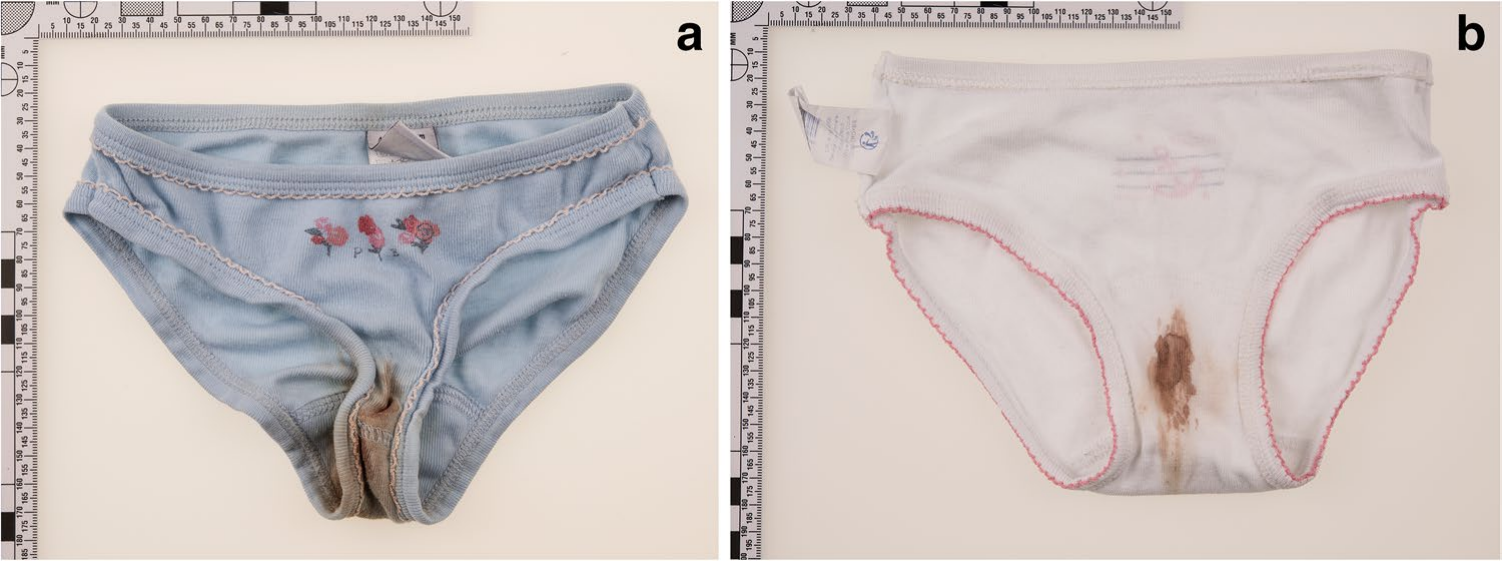
Bloodstains in the girl’s panties. **a** Bleeding at day 1. The bleeding soaked several layers of clothing. **b** Persistent bleeding on day 2

The mother of a 5-year-old girl of African origin (Gabon) noticed bloodstains in the girl’s panties (Fig. 1), and suspected vaginal bleeding. As the bleeding continued the next day, and as there was no trauma reported and no conclusive explanation, the mother presented the girl at the pediatric emergency room. At the clinical examination, the physician detected an obvious abnormality of the external genital organs of unknown origin, and interpreted the findings as traumatic. After excluding an intravaginal foreign body by transabdominal ultrasound, there was suspicion of sexual abuse, and the police were involved.

During the questioning by a police officer, the girl kept repeating that itching made her scratch her genitalia, without mentioning any other manipulation. The mother explained that her husband (the father of the girl), her 2-year-old sister and three brothers, among them two adolescent half-brothers, lived together in one household. The mother stated that the girl was under her supervision the whole day except for a period of 15–30 min when the girl was in the bathroom.

Subsequently, the magistrate ordered a medicolegal opinion, and a further gynecological and a clinical forensic examination were initiated.

### Case 1: results of the gynecological and clinical forensic examination

**Fig. 2:**
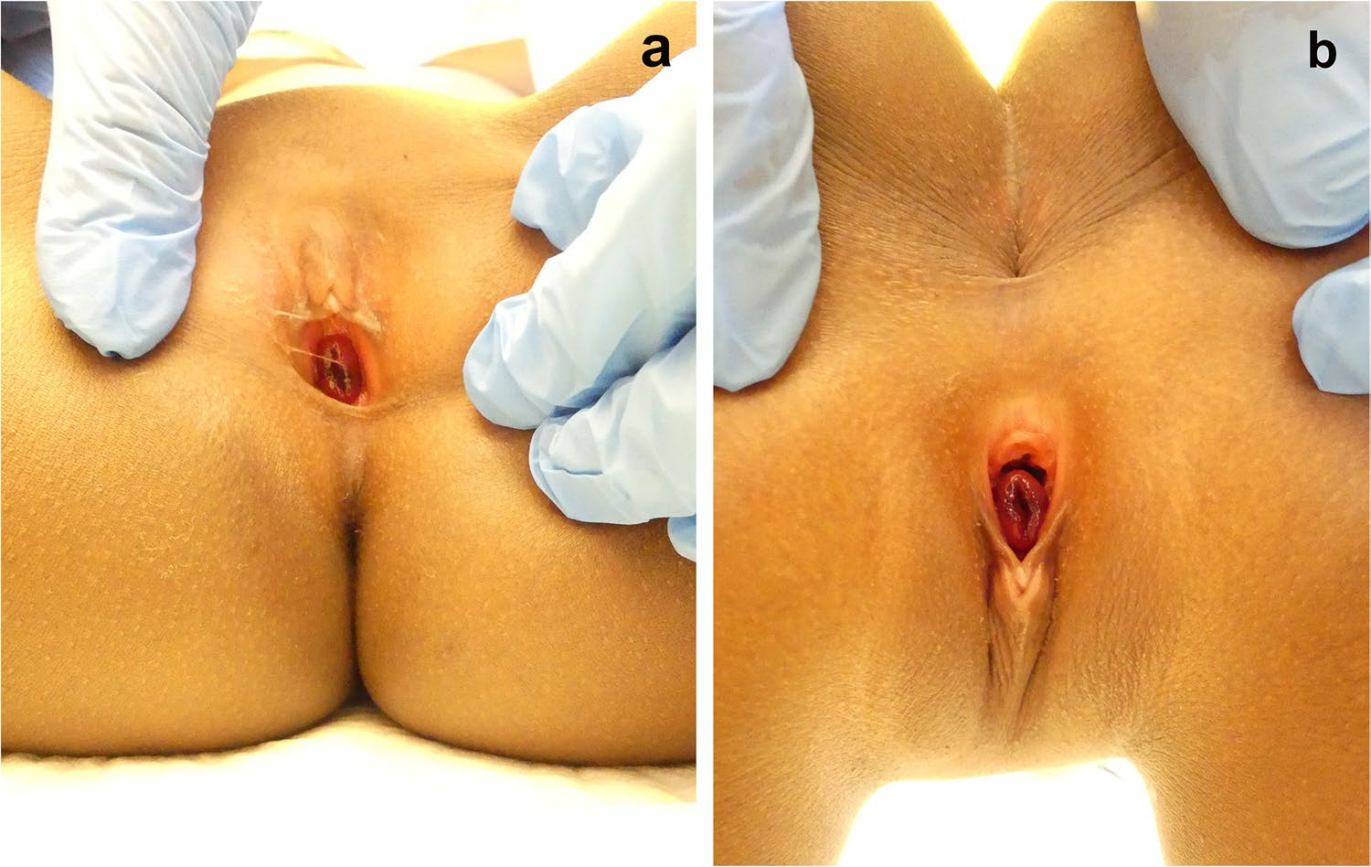
Findings of the gynecological examination in two different positions (case 1). **a** Supine position; **b** knee-chest-position. Note the doughnut-shaped, prolapsed tissue masking the vaginal entrance in supine position; the knee-chest position is more suitable to visualize the vaginal entrance and thus to facilitate the correct diagnosis

At the time of the examination, the girl appeared neither physically compromised nor emotionally affected. The external genital organs were inspected in both supine and knee-chest position. At the vaginal entrance, prolapsed tissue of a doughnut-like form and livid coloration, easily bleeding on contact, could be observed (Fig. 2). The hymen was not visible due to the mass occupying the vaginal entrance and masking other structures. Swaps for DNA analysis and infectious diagnostics were collected. The physical examination revealed no other particularities, especially no injuries and no signs of physical abuse, hormonal disorder, or premature puberty.

### Case 1: further examinations, diagnosis, and outcome

Microbiological diagnostics were negative for urogenital infections and sexually transmitted diseases. After a literature search, the suspected diagnosis of a urethral prolapse was made and confirmed by a specialist based on the pictures taken during the examination. The treatment was conservative, consisting of sitting baths and topical application of estrogen. The patient was seen for regular controls in pediatrics ambulatory care. After another period of bleeding in the first days, the response to the treatment was finally satisfactory. No complications, e.g., dysuria, pain, anemia, or urinary retention, occurred, and no further treatment was necessary.

### Case 2: history

A 7-month-old girl of South European origin with a history of congenital macrocephaly and exophthalmia presented at the hospital for a medical specialist’s opinion and further examinations. At the general clinical examination, the physician detected a wet linear laceration of the perineum that had not been diagnosed before. The lesion did apparently not bother the child, who was under the care of both parents. The mother stated that she had not noticed this finding before and that there had never been a genital bleeding. Equivocal statements of the parents and an apparently difficult social setting further raised the suspicion, of a sexual abuse among the health-care professionals, and the case was reported to the prosecuting authorities. The magistrate in charge of the investigation ordered a medicolegal opinion and a clinical forensic examination was arranged.

### Case 2: results of the gynecological and clinical forensic examination

**Fig. 3:**
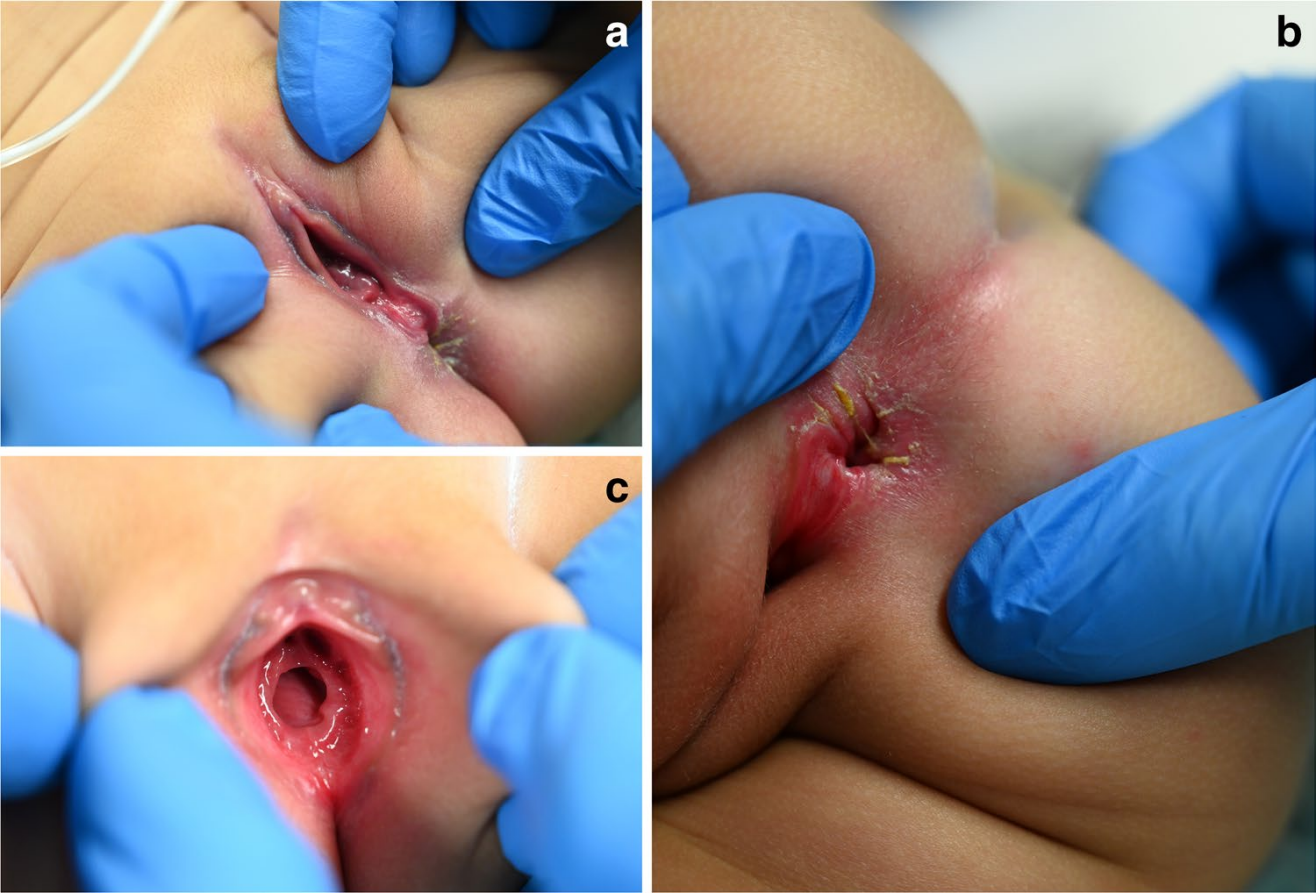
Findings of the gynecological examination in two different positions (case 2). **a** Supine position; **b**: lateral position. Note the complete perineal groove extending in the midline from the posterior fourchette to/into the anus. **c** Visualization of the hymen. Note that the hymen showed no particularities, especially no signs of penetration

Ten days after the initial examination, the girl underwent an MRI under sedation for her underlying cerebral disease. The opportunity allowed for a gynecological and clinical forensic examination under optimal conditions. After separation of external labia in supine position, a wet unkeratinized perineal defect in extension of the internal labia with well-demarked raised margins was detected in the midline of the perineum (Fig. 3a). Apart from this defect, the external genital organs were normal. The hymen visualized in supine frog-leg position was without any particularities (Fig. 3c). In the lateral position, the consistently superficial lesion appeared to extend continuously to the anus (Fig. 3b). There were no bruises and no signs of tissue repair or infection. The anus seemed anteriorly placed but had a normal appearance. The physical examination furthermore revealed an obvious hypertelorism, exophthalmos, low-set ears, and a Mongolian spot on the lower back. There were no injuries and no signs of physical abuse or child neglect.

### Case 2: further examinations, diagnosis, and outcome

In order to clarify whether the perineal defect was recent or known to be congenital, the police officer in charge of the investigation contacted the resident pediatrician, who had performed the routine check-ups of the girl. Unfortunately, the anogenital region had not been inspected in detail prior to the hospital admission and the age of the lesion remained unclear.

The morphological appearance of the defect—absence of typical signs of injuries like an irregular configuration and hematoma/ecchymosis, and the lack of healing of the lesion between the first and the second examination (when compared to photographs that were taken during the first examination)—made it seem unlikely to be due to trauma. A literature search lead to the diagnosis of a congenital failed fusion of the perineum known as perineal groove. Given the good prognosis of this pathology and the fact that there were no symptoms and no complications, no special treatment was necessary. A genetic survey was initiated to rule out a possible genetic cause of the syndrome. The further treatment of the underlying disease and the follow-up are unknown.

## Discussion

We report on two cases of young girls with no history of recent trauma presenting suspected vaginal bleeding and an apparent lesion of the perineum, respectively. Both findings were initially misdiagnosed as sequels of sexual violence, and led to police investigations. The underlying diseases, namely a urethral prolapse and a failure of the midline fusion of the perineum, are differential diagnoses of sexual abuse [4]. However, these pathologies are rarely seen in pediatric clinical practice, and, in consequence, are frequently misdiagnosed. Ninomiya and Koga [5] reported a rate of correct diagnosis on initial examination of only 21% for urethral prolapse and Samuk et al. [6] of 9% for perineal groove. Reliable data on incidence are not available, and only few cases have been reported in the forensic literature.

As the perineal groove is a congenital malformation that usually resolves spontaneously during the first two years of life [6], it is seen in newborns as well as in young children with no apparent predominance to ethnic origin. Urethral prolapse on the other hand is occurring in young prepubertal girls aged predominantly between 4 and 8 years, and mainly affects girls of African origin [5, 7]. The exact pathogenesis and potential risk factors of the perineal groove remain uncertain. The urethral prolapse is related to increased abdominal pressure (e.g., constipation, chronic coughing), and a lack of estrogen (known to be responsible for urethral prolapse in adults) is also postulated for children [8].

Urethral prolapse almost exclusively affects girls, whereas perineal groove seems somewhat more likely to appear in males. To our knowledge, three cases in males have been published so far [6]. A combination with other, more obvious malformations of the genital organs, such as hypospadia, which is seen in two of three cases involving boys with a perineal groove [6], makes misdiagnosis as an injury in males less likely.

Thus, a context of dysmorphic stigmata, as presented in the perineal groove case, allows for the physician to take into consideration a congenital malformation rather than an injury when confronted unexpectedly to such a rare genital condition. However, a literature review shows that such a context is rather uncommon in girls: most cases published did not show other external abnormalities, and only a minority of them was combined with internal congenital malformations like persistent ductus arteriosus [9], or co-existing anorectal malformations [6].

The later the defect is discovered, the more likely it is to be mistaken for an injury. Samuk et al. [6] observed a time span of diagnose delay from 1 day to 58 months. Delayed diagnosis of perineal groove may be explained by the fact that the defect is only visible when the buttocks are separated. Furthermore, as the condition usually does not lead to symptoms, caregivers are often not as concerned as they would be if the child was experiencing obvious symptoms, such as bleedings.

Urethral prolapse, in contrast, shows bleedings as most frequent symptom (86%), followed by a mass at the introitus (47%) and dysuria (32%) [5].

Lower genital tract bleeding in a child is an alarming symptom for parents (or other caregivers), and usually leads to medical help being sought immediately. Adams et al. [10] classified lower genital tract bleeding as a reliable predictor for abnormal findings in the pediatric gynecological examination. In the case of urethral prolapse presented, the large bloodstains in the girl’s panties (Fig. 1) illustrate how striking the manifestation can be. Relatives and health-care professionals tend to relate this symptom to acute trauma, and suspicions of recent sexual violence, primarily penetration, frequently emerge, especially when there is no history of accidental trauma. In contrast to the “myth” that (vaginal) penetration is one of the main reasons for vaginal bleeding in children, Zhang et al. [11] showed that predominant causes for vaginal bleeding in girls aged from 10 month to 10 years are foreign bodies, followed by vulvovaginitis and accidental vulvar trauma. Kelly et al. [12] report that physical symptoms such as vaginal bleeding were of no value in discriminating sexually abused children from those who had not been abused; and Russo et al. [13] pointed out that even bleeding from injuries after sexual violence could be delayed by several hours.

A comprehensive and meticulous gynecological and physical examination should usually reveal the respective lesion. For urethral prolapse, recognizing its origin from the external meatus of the urethra is essential to establish the diagnosis. However, the mass may be confounded as the vaginal entrance, since the protruding tissue frequently masks the normal anatomy of the external genital organs, resulting in a high rate of misdiagnoses. Detecting the vaginal entrance beside the prolapsed tissue may help determine the origin of the prolapse. In supine position, gentle posterior traction of the external labia can uncover the vaginal entrance. If the protruding tissue mass is voluminous, it is more likely to get to see the vaginal entrance in knee-chest position than in supine position. Putting a catheter through the center of the mass and drain urine can be necessary if there are voiding disorders, and may help to confirm the diagnosis.

In order to differentiate perineal groove from traumatic lacerations of the perineum, it is essential to focus on the morphology of the lesion. The perineal groove extending in the midline from the posterior fourchette to the superior anal verge is equal in depth, clearly delimited with regular margins, and painless, whereas lacerations from trauma tend to be deep, bruised, and painful. Another particularity of the malformation is the absence of visible wound healing. Injuries heal rapidly, often ad integrum, within days to weeks, whereas the spontaneous epithelialization of a perineal groove usually takes at least several months, in some cases even years [6, 9, 13]. An incomplete—anterior (15.3% of the cases) or posterior (30.7%) [6]—perineal groove may be even more difficult to recognize.

Both the perineal groove and the urethral prolapse have in common that the diagnosis is made clinically and that the treatment can be conservative or expectant, respectively, except if there are complications or persistent complaints [7, 9, 14].

In the literature [15], pelvic trauma and rape are occasionally mentioned among the potential risk factors for urethral prolapse, but to our knowledge, no case of confirmed sexual abuse presenting with urethral prolapse as leading symptom has been published so far. Nevertheless, the confirmation of a urethral prolapse as well as a perineal groove cannot per se exclude sexual abuse. Therefore, in suspected cases of child sexual abuse, the gold standard is a multidisciplinary approach involving an experienced child gynecologist and an expert in clinical forensic medicine, to take into account different indicators, and to follow existing recommendations and standards. Awareness of and knowledge about the morphology and clinical presentation of rare diseases such as urethral prolapse and perineal groove are indispensable, and should be enhanced among health-care professionals and forensic experts. A skilled eye aware of the existence of these pathologies and excellent examination conditions will in the end lead to a correct diagnosis.
